# Remarkable Case of Strangulated Hernia

**Published:** 1816-12

**Authors:** William Henry Carpenter

**Affiliations:** Member of the Royal College of Surgeons, London.


*For the London Medical and Physical Journal.*

### *Remarkable Case of Strangulated Hernia;* by WILLIAM HENRY CARPENTER, Member of the Royal College of Surgeons, London.

CONCEIVING that the relation of cases is essentially important to the medical practitioner, I am induced to offer the following, as an instance in which the powers of nature proved sufficient, where the aid of the knife could not have been safely or judiciously employed.

On July the 27th, late in the evening, I was requested to visit Richard Litley, of this town, æt. 58; and, on my arrival, found him evidently labouring under the symptoms of a strangulated hernia. In answer to my enquiry, I was informed that he had been subject to an irreducible rupture from his childhood; and, upon examination, I found one of the largest scrotal hernias I ever recollect seeing. On further enquiry, his friends informed me that he had, the day previous to my seeing him, lifted a large quantity of faggot wood,—to which they attributed his present complaints, as the hernia had increased much in size from that period. He complained of pain in the region of the abdominal ring, accompanied with flatulency; had vomited several times; the swelling was very tense, and the integuments a good deal inflamed; his pulse was hard and quick. I immediately took, from a large orifice in the arm, thirty ounces of blood, and then had recourse to the taxis, in hopes of being enabled to effect a reduction of the strangulated portion; but found my attempts fruitless, from the tender state of the tumour, accompanied with considerable inflammatory action. I then ordered ordered him some laxative medicine, promising to see him again early the following morning, which I did, and found him still unrelieved; but the symptoms were not sufficiently violent to warrant the necessity of an operation. The medicine which he took the preceding night not having operated, I ordered him some cathartic extract, and a laxative mixture, consisting of infusion of senna, with the tincture and sulphate of magnesia, to be taken every two hours; directing the part to be kept constantly cold, with a lotion made with equal parts of nitrate of potass and muriate of ammonia.

At eight P. M., finding the symptoms rather increased, I again took from the arm thirty more ounces of blood, and attempted the application of the taxis, but without success. The medicine which he took in the course of the day not having operated, I requested that a common enema might be thrown up.

On the 29th, at eight P. M. I found him worse than when I left him the previous night. He now expressed a wish to have more blood taken away, as he always appeared relieved by the former bleedings. I drew, from a large opening in the other arm, twenty-four more ounces, and gave him one ounce of ol. ricini; the enema to be repeated, as the other had not operated.

At six P. M. the same day, I found his symptoms assumed a more aggravated form: tension of the abdomen had taken place, accompanied with considerable pain and difficulty of breathing, with tightness across the region of the diaphragm. As I had failed in all my former attempts in procuring evacuations, I now deemed it proper to have recourse to the tobacco enema as my last remedy. Infused ʒj. of tobacco in a pint of boiling water, and threw up half the quantity. It immediately produced that debilitating effect upon the arterial and nervous system which I had always observed in previous cases of strangulated hernia, and, indeed, so much so, that I was apprehensive, for some time, my patient could not long survive its sedative influence. In less than ten minutes, however, he had an evacuation; and, upon examination, I found that all the clysters which had before been given were come off, mixed with a portion of fæculent matter; but a doubt still existed in my mind whether or not it had passed beyond the strictured part: he certainly appeared relieved at the time, and I left him with the hope that more evacuations would follow in the course of the night.

On the 30th, at nine A. M., I again saw him, and was informed that he had had no motions since the one which followed the tobacco injection; from the effects of which he had, had, in some degree, recovered; but his symptoms continued to increase considerably, and I saw no other chance left but the performance of an operation. In the course of the day, I requested the opinions of two other medical men, who immediately agreed with me that it was the only thing which could afford a probability of relief. The man readily consented to my proposal; and in the evening I proceeded to perform it, in the presence of those gentleman whom I had before consulted. I began my incision at the external ring, extending it half way down to the bottom of the scrotum, and, having divided the different coverings, cautiously made a small opening into the sac, which I then dilated. A large portion of intestine immediately protruded, the appearance of which was not so unfavourable as I had anticipated. I then searched for the stricture, which I found situated at the outer ring; and, having divided it, I then attempted to return the intestine, (there being very little omentum down,) but found that adhesions had taken place to the sac, so very extensive, that all our endeavours were impracticable, and to attempt the division of them with the knife would greatly endanger the safety of my patient. Being thus foiled, with the concurrence of those around me, I attempted to bring the divided integuments over the exposed gut; but here we were also equally disappointed, as the tense state of the skin would not permit its being brought in contact. In this condition, we were obliged to place the patient in bed, covering the exposed intestine, which was very extensive, with some dry lint. It was my opinion, as well as the opinion of my medical friends, that he could not long survive the exposure of so large a portion of intestinal surface. We left him.

Early the next morning I again saw him, and was pleased to find that, in the course of the night, he had procured an evacuation, and appeared evidently relieved by the operation, although he still laboured under much pain and constitutional irritation. There was considerable tension in the right side of the abdomen, and the slightest pressure gave much pain. The appearance of the intestine was good; and the discharge considerable. I ordered an enema, which had the effect of producing more motions, and he appeared better in consequence. From that time he gradually recovered; and the symptoms of strangulation daily diminished. The wound assumed a disposition to heal; and, in a short time, the protruded intestine gradually contracted within the boundaries of the divided scrotum, which was followed by cicatrization; and, by the 1st of October (two months from the operation), the parts were completely healed, and the the patient enabled to resume his usual occupation. The dressings consisted of lint and strips of adhesive plaster;—occasionally some laxative medicine were found necessary.

*Ottery St. Mary, Devon;*
*Nov.* 4, 1816.

P.S.—It may be necessary for me to assign some reason why I made so extensive an opening into the sac, previous to the performance of the operation. It was my opinion that the adhesions were numerous, as far as it regarded that portion of intestine which had been down so many years; but I was in hopes of being enabled to return that which had so recently descended, by doing which I should have had integuments sufficient to cover the remainder.

---

*** We take the liberty to suggest to Mr. Carpenter the probability of the above having been a congenital hernia. See Mr. Wadd's ingenious chapter on that subject in his "Cases of Diseased Bladder and Testicle."—EDIT.

